# Mitral Stenosis

**Published:** 1899-09

**Authors:** M. F. Carson

**Affiliations:** Griffin, Ga.


### MITRAL STENOSIS.*

BY M. F. CARSON, M.D.,

GRIFFIN, GA.

Stenosis of the mitral orifice is a condition which is often easily diagnosed at the bedside; but nevertheless there are many cases in which its recognition is difficult, if not impossible, and one finds more frequently difference of opinion as to whether mitral constriction is or is not present, than one does as to the presence or absence of any other chronic valvular defects.

Anatomically the results of the gradual thickening and constriction of the mitral valve are reached in the well-known conditions the "button-hole" orifice and the "funnel-shaped" orifice. The former generally occurs when the flaps of the valve are pretty uniformly affected, so that the general shrinkage produces an oval or sometimes roundish orifice in the thickened curtain. In the case of the funnel-shaped orifice the free edges of the valve are disproportionally thickened and the flaps still jut out into the left ventricle, where the apex of the funnel is seen, while the insertion of the valve in the wall of the heart forms the larger extremity of the funnel.

But such anatomical details are not to be made out ordinarily, and the best physicians are content with the diagnosis of mitral constriction.

When one examines a well-marked case of the disease, we notice on inspection that the apex beat of the heart is in its normal situation, the meaning of this being that the left ventricle is not enlarged; indeed, it may be diminished in size. This depends upon the fact that its work is lessened owing to the auricle not being able to discharge so much blood into it, as it does in health.

But this only applies to cases where there is no regurgitation, that is, to cases of pure mitral constriction. In mitral regurgitation the auricle, which is over-filled at great pressure by the blood poured into it through the mitral orifice, discharges its contents

* Read before the State Medical Association at Macon, April, 1899.

again into the left ventricle with accumulated force, as soon as the ventricle ceases to contract; and the latter hypertrophies to meet the abnormal strain.

On palpation a thrill is felt at the end of the diastole, which is brought to an abrupt ending by a short, sharp cardiac impulse. The ventricle, receiving a small quantity of blood, can contract more readily than under normal conditions, and thus the short, sharp apex beat is produced.

On percussion, the cardiac dullness is found to be normal towards the left, and generally increased towards the right, reaching sometimes nearly as far as the right nipple. It may also extend upwards on the left of the sternum to the third, or even to the costal cartilage. These changes are due to an increase in the size of the left auricle, right auricle and right ventricle, an increase which depends partly on dilatation, partly on hypertrophy.

Auscultation yields pretty definite and constant results in most valvular diseases.

In aortic regurgitation, and in mitral regurgitation, for example, the murmurs heard in successive cases are not very different. But in mitral constriction it is far different. The observer may hear:

1. A presystolic murmur; that is, a murmur which occupies the last third or half of the diastolic period only. Two conditions are essential to the production of the presystolic murmur. The first is a sufficiently narrowed orifice, so that in the earlier stages of constriction no murmur is produced. The second is a sufficiently high blood pressure in the auricle, so that the stream of blood may be driven with force enough from the auricle into the ventricle to give rise to vibrations and produce the murmur. It may be that as the blood passes through the mitral orifice the stream is not forcible enough to produce any sound until it is accelerated by the contraction of the auricle. It is under these circumstances that the presystolic murmur is heard.

2. But there is another period in the diastole in which the blood pressure in the auricle may be great enough to produce a murmur, and that is at the commencement of diastole. The mitral orifice being narrowed, the left auricle becomes distended with blood, and when the ventricular systole occurs it stops the inflow of the blood and still further heightens the pressure in the auricle. As soon as the contraction of the ventricle is over, the blood flows onward under very high pressure, and may give rise to an early diastolic murmur. As far as my experience goes, this murmur rarely exists alone. When it is present it runs into the presystolic murmur, and consequently a murmur is heard continuously from beginning to end of diastole. But in these cases the murmur, as a rule, becomes louder and more rumbling during the presystolic period. This point is of some importance in diagnosis, because a continuous even murmur, occupying the whole diastole, and produced at the mitral orifice, may be due to a dilated and not to a constricted opening.

3. In some cases, or at least at certain periods of some cases, a continuous murmur, uniform in character, may be heard during the whole of the diastole. The reason of this probably is, that the narrowing of the orifice, and the force of the accumulated blood behind, are sufficient to give rise to such a murmur; but the auricular contraction is so enfeebled as to be unable to produce much acceleration of the stream in the presystolic period.

4. There may be no murmur. This may not only be the case in the earlier stages of constriction, but in the latter also. The presystolic murmur, depending on auricular constriction, is loud when the latter is energetic, and may totally disappear when the auricle fails, and may reappear again when the auricle recovers power.

Besides the murmur, some other points are of importance in auscultation. One is, that in a great number of cases of this disease the sound is not heard at the apex.

Another is, that the first sound is short and sharp, owing probably to the ease and suddenness with which the left ventricle can contract upon its diminished contents.

A third point is, that the second over the pulmonary valves is accentuated. In doubtful cases of mitral stenosis this is of the utmost importance, especially where there is no other condition discoverable which could explain this phenomenon.

There is one other auscultatory sign which, in the absence ot others, suggests to a careful observer the presence of the disease. It is a peculiar hesitation in the production of the first sound, a kind of hanging fire, which is easier to appreciate with the stethoscope than to describe. This may be due to the prolongation of the diastole, for the blood goes through the mitral orifice slowly, and it takes a somewhat longer period of time to produce the degree of blood pressure in the left ventricle which gives rise to its contraction.

*The Course of the Disease.*—This may be divided into two periods; the first, that of good compensation; the second, that of bad, failing compensation. During the first of these periods there is a moderate degree of the narrowing of the orifice, and auricle is not only dilated, but also hypertrophied. There may then be very few symptoms, and the disease may only be discovered, so to say, accidentally, that is to say, during the routine examination of a patient's organs who makes no complaints with reference to the heart; or there may be some shortness of breath on unusual exertion; or slight blueness of the lips and cheeks. The pulse is generally small and may be very infrequent indeed. Well-marked bradycardia may be present in mitral stenosis, the number of beats per minute being as low as forty. In other cases the pulse-rate may be quickened.

In the second period, that of failing compensation, the auricular walls become enfeebled, and the cavity of the auricle dilated, and the blood accumulates more and more behind the orifice, in the lungs, right heart, and general venous circulation. Then the pulse becomes small, feeble, rapid, and irregular, and many of the heart's beats, as many even as half, may produce no appreciable effect upon the radial artery. The frequency of the pulse then can only be safely estimated by auscultation of the heart. This fact is not always borne in mind, and the notes of such cases sometimes give a very inaccurate record of the frequency of the heart's beat.

The blood being blocked in the lungs, respiration becomes more rapid, and dyspnea or even orthopnea occurs, accompanied by more or less cyanosis. Fluid may ooze from the distended pulmonary capillaries, causing edema of the lungs, or hemorrhagic infarction with hemoptysis may occur.

The latter occurrence is ordinarily the result of embolism of one of the peripheral pulmonary arteries by the dislodgment of a piece of thrombus formed in the cavities of the right side of the heart; but it probably also originates at times from primary thrombosis in the vessels of the lungs.

The obstruction of the general circulation is shown by dropsy, occurring first in the feet, and later more universally. Before this occurs, however, the failure of the right side of the heart is often rendered evident to the careful observer by the enlarging and tender liver, and the examination of this organ is very important in chronic valvular disease, even from a prognostic point of view.

The diminished arterial pressure, and the stoppage of the free flow of blood through the kidneys, produces a diminution, often great, in the quantity of urine passed, which is concentrated and high colored. It may also contain albumen and many casts. The latter are, however, hyaline, not granular or cellular, and only indicate obstruction in the renal circulation and not organic disease of the kidneys. The microscopic examination of a few kidneys in persons who have died from cardiac dropsy has convinced me that mere venous congestion of these organs from heart disease does not give rise to serious organic disease, such as contracted kidney. Nor do I believe that the so-called "nutmeg" liver ends in cirrhosis. It is true that the connective tissue of these organs in chronic cases may look hard in outline and a little thickened, but serious cirrhosis of the liver or kidneys does not result from venous obstruction.

The latter, it must be remembered, may often occur in patients who already have cirrhosis from other causes. The spleen from mitral constriction is sometimes large and sometimes small, always hard, and may show areas of recent or old infarction, as also may the kidneys. When sudden infarction occurs in the latter organ, it is generally evidenced at the bedside by hematuria with or without lumbar pain, and cellular and granular casts may appear in the urine.

The altered circulation in the gastro-intestinal canal frequently causes troublesome dyspepsia, and sometimes hemorrhage from the bowels; while the obstruction in the cerebral circulation may produce serious insomnia, and sometimes delirium.

Or a fibrinous embolus from the left auricle blocks one of the cerebral arteries, and may produce limited or wide-spread softening.

In such cases of general dropsy it now and then occurs that some large vein, as the axillary or femoral artery of one side becomes obstructed by a local thrombus, and then the swelling, pain, and tension in the corresponding limb are much greater than elsewhere. A patient, too, who can only lie on one side may thus get an unsymmetrical dropsy, owing to gravitation.

Certain physical signs may occur in connection with the heart when the stage of failure sets in, which are often of importance in estimating the degree of failure which has occurred, or in foreshadowing its occurrence. Thus, the presystolic murmur may disappear, owing to the complete or partial failure of the contraction of the left auricle, and a sign of similar import is the diminishing intensity of the second sound on the pulmonary side. The more complete the stasis in the pulmonary circulation and the left auricle the less the quantity of blood which flows through the pulmonary orifice at each contraction of the right ventricle.

The semilunar valves therefore undergo less movement, less sudden change from flaccidity to tension, and the sound they produce diminishes in intensity. In the stage of failing compensation the left ventricle may gradually hypertrophy, as the tension in the arteries, which it has to overcome, increases, owing to backward pressure. The appearance of a tricuspid murmur, and pulsation in the jugular vein, or even in the liver, may testify to a dilated tricuspid orifice allowing of regurgitation.

The patient, whose distress increases from so many causes, may gradually die in spite of every effort we may make to save him.

On the other hand the treatment of such cases is, as a rule, very hopeful, and although the actual narrowing of the mitral orifice must still remain and may even increase, death may be averted and years of fairly comfortable and useful, though quiet, life be secured. Under favorable circumstances, the dropsy gradually disappears, the urine increases in quantity, and albumen and casts disappear, the liver becomes smaller and less tender. Respiration becomes easier, the pulse slower, more regular and fuller, the presystolic murmur reappears, and the second pulmonary sound becomes louder. The patient sleeps better, eats and digests his food better, and gradually returns to a condition of comfort.

*Treatment.*—The first and most essential point in the treatment of heart failure in cases of valvular disease is rest; the patient should be put to bed. This measure of itself sometimes gives wonderful results. If the venous congestion is very great, bleeding should be resorted to in mitral stenosis.

As regards drugs, digitalis still holds the foremost place where the pulse, is rapid, feeble and irregular, and in many cases its effects are very striking. But I believe it is useless to give digitalis with a slow pulse, that is, where the pulse is beating at a rate which is normal to the individual in question, or below normal. We cannot hope to increase the force of the circulation as a whole, if, while we add to the power of each contraction of the heart, we diminish the number of beats per minute; unless, indeed, when we begin to give digitalis the heart's beats are abnormally hurried as well as feeble.

Under such circumstances digitalis sometimes fails. Strophanthus may then be tried; but the latter drug is far less frequently effectual than digitalis. Combined with digitalis we may give strychnine, which I believe is one of the best, if not the best, cardiac tonics we possess if given subcutaneously.

It may be given when the heart's action is slow, and it may be sometimes after digitalis has reduced the frequency of the pulse. Another very favorite drug with me, especially if the urine is scanty, is citrate of caffein. I believe alcohol should be given in moderate quantities.

The bowels should be kept well open, but in my experience purging is not advisable. Dyspepsia generally depends on the congestion of the liver and gastro-intestinal mucous membrane, and is relieved by the remedies which improve the heart's action; in this condition I have used bicarbonate of soda with some bitter infusion with fairly good results.

Abundant simple food in small quantities, often repeated, is most essential in the successful treatment of this condition.

I have found that one of the most difficult complications to manage is insomnia. Morphia by the mouth or in small quantities subcutaneously often acts well. Small doses of chloral can be given with caution, and are best given with alcohol. In my hands sulphonal and many others of the much-lauded new narcotics have failed.

